# Development and Psychometric Validation of the Geriatric Patient Safety Culture Questionnaire for Intensive Care Nurses

**DOI:** 10.3390/healthcare14203473

**Published:** 2026-10-16

**Authors:** Eman Mahmoud Mohammed Shoukr, Haitham Mokhtar Mohamed Abdallah, Mohamed E. H. Elzeky, Mahmoud Abdelwahab Khedr, Abeer Abd-El Rahman Mohammed, Heba Ahmed Mohsen Hassen, Wafa Hamad Almegewly, Mazen Baazeem, Noha F. M. Shahine

**Affiliations:** 1 Gerontological Nursing Department, Faculty of Nursing, Alexandria University, Alexandria 21526, Egyptabeer.mohamed@alexu.edu.eg (A.A.-E.R.M.);; 2 Medical Surgical Nursing Department, College of Nursing, Jouf University, Sakaka 72388, Saudi Arabia; 3 College of Nursing, University of Hafr Albatin, Hafr Albatin 39524, Saudi Arabia; 4 Department of Community and Psychiatric Mental Health Nursing, College of Nursing, Princess Nourah bint Abdulrahman University, P.O. Box 84428, Riyadh 11671, Saudi Arabia; 5 Department of Health Information Management and Technology, College of Applied Medical Sciences, University of Hafr Albatin, Hafr Albatin 39524, Saudi Arabia; 6 Gerontological Nursing Department, Faculty of Nursing, Mansoura University, Mansoura 35516, Egypt

**Keywords:** critically Ill geriatric patients, safety culture, intensive care nurses, psychometric validation

## Abstract

**Background:** Critically ill geriatric patients represent a unique group with specific care needs due to the physiological and pathological changes associated with aging, which make them susceptible to adverse events and complications during their stay in the Intensive Care Units. This study aimed to develop and psychometrically validate the Geriatric Patient Safety Culture Questionnaire (GPSC-Q), which assesses ICU nurses’ knowledge, attitudes, self-reported practices, and perceptions of the work environment related to the safety of critically ill geriatric patients. **Method:** A methodological study was conducted among 330 ICU nurses to develop and evaluate the psychometric properties of the multidimensional GPSC-Q. The psychometric evaluation included content validity, exploratory and confirmatory factor analyses, internal consistency, and convergent and discriminant validity assessments. **Results:** The final questionnaire comprised 43 items: 15 Knowledge, 7 Attitude, 12 Practice, and 9 Work Environment items. Exploratory factor analysis identified four, one, four, and three factors, respectively. Internal consistency coefficients were 0.902, 0.936, 0.749, and 0.721, respectively. Knowledge and Practice showed satisfactory primary CFA fit, whereas Attitude showed mixed fit, including an elevated RMSEA of 0.083. Work Environment showed weaker fit (CFI = 0.907, TLI = 0.855, RMSEA = 0.089) and lower reliability and convergent validity in two domains. The second-order CFA showed mixed fit (CFI = 0.936, TLI = 0.930, RMSEA = 0.048, SRMR = 0.070), with CFI and TLI below the prespecified criteria. **Conclusions:** The GPSC-Q provides preliminary psychometric support for assessing ICU nurses’ knowledge, attitudes, self-reported practices, and perceptions of the work environment related to geriatric patient safety. However, support varied across subscales, with weaker findings for Work Environment. Further refinement and validation in independent samples are needed before recommending broad application. **Implications for Practice:** The GPSC-Q provides a structured approach to assessing nursing and work environment dimensions relevant to geriatric patient safety in intensive care. Further validation may support its use in identifying educational priorities and informing organizational safety initiatives.

## 1. Introduction

Across the globe, countries are experiencing a rise in aging populations, which is shifting hospital demographics toward an older patient base. This trend is leading to growing pressure to adapt healthcare services to better meet the needs of older adults [1]. Over the past two decades, more elderly individuals have been admitted to intensive care units (ICUs). In many nations, the median age of critically ill patients is now nearing 65, and the number of geriatric patients aged 80 and above is increasing more rapidly than any other age group in ICUs [2].

Older adults are more likely than younger patients to suffer from adverse events (AEs) and preventable AEs while hospitalized, particularly in intensive care units (ICUs). Research from the Netherlands showed that 2.9% of hospitalized geriatric patients experienced a preventable AE, compared to 1.8% of younger patients [3]. An adverse event refers to unintended harm that leads to temporary or permanent disability, death, or a longer hospital stay, and is linked to the delivery of healthcare, not the patient’s underlying condition. When such events result from a failure to follow standard procedures, they are considered preventable [4]. In Spain, Merino et al. found that 41.1% of ICU patients in a teaching hospital experienced at least one AE, and 70% of those were preventable [5].

Strengthening patient safety culture (PSC) is an important priority for improving the quality and safety of intensive care [6]. Critically ill geriatric patients have complex care needs associated with age-related physiological changes, multimorbidity, and the severity of acute illness [7]. Their care also involves ethical and practical challenges, including admission decisions and communication with families and caregivers [8]. These considerations require attention to both geriatric-specific nursing care and the organizational conditions supporting its safe delivery. Assessing PSC helps identify strengths and areas requiring improvement and informs efforts to reduce avoidable harm [9,10]. Patient safety culture encompasses the shared values, beliefs, norms, and organizational arrangements that shape how safety is prioritized and enacted. Safety climate refers more specifically to staff perceptions of the priority given to safety through organizational policies, procedures, and everyday practices. Although related, these concepts are not interchangeable. A positive safety culture is characterized by trust, leadership commitment, teamwork, and systems that support reporting and learning from errors [9,10]. In geriatric care, assessment should also consider whether care processes and work environment conditions support patients’ specific safety needs [11].

Established instruments, including the Safety Attitudes Questionnaire (SAQ), the Patient Safety Climate in Healthcare Organizations survey (PSCHO), and the Hospital Survey on Patient Safety Culture (HSOPSC), assess important organizational dimensions, including teamwork, safety climate, management support, and working conditions [12,13,14,15,16]. These instruments remain useful for assessing safety culture in settings caring for older adults. However, their general organizational focus does not provide the geriatric-specific assessment of nursing knowledge and reported care practices required for the present study. Critically ill older adults have safety needs associated with age-related physiological changes, multimorbidity, and the complexity of intensive care [7,8,17]. Relevant nursing care issues include medication safety, skin integrity, mobility and fall prevention, mental status assessment, nutrition, and restraint use. The assessment gap is therefore the need to examine geriatric-specific knowledge, attitudes, and self-reported safety practices alongside work environment conditions within a single questionnaire for ICU nurses. Such an instrument would complement established safety culture measures.

**Figure 1:**
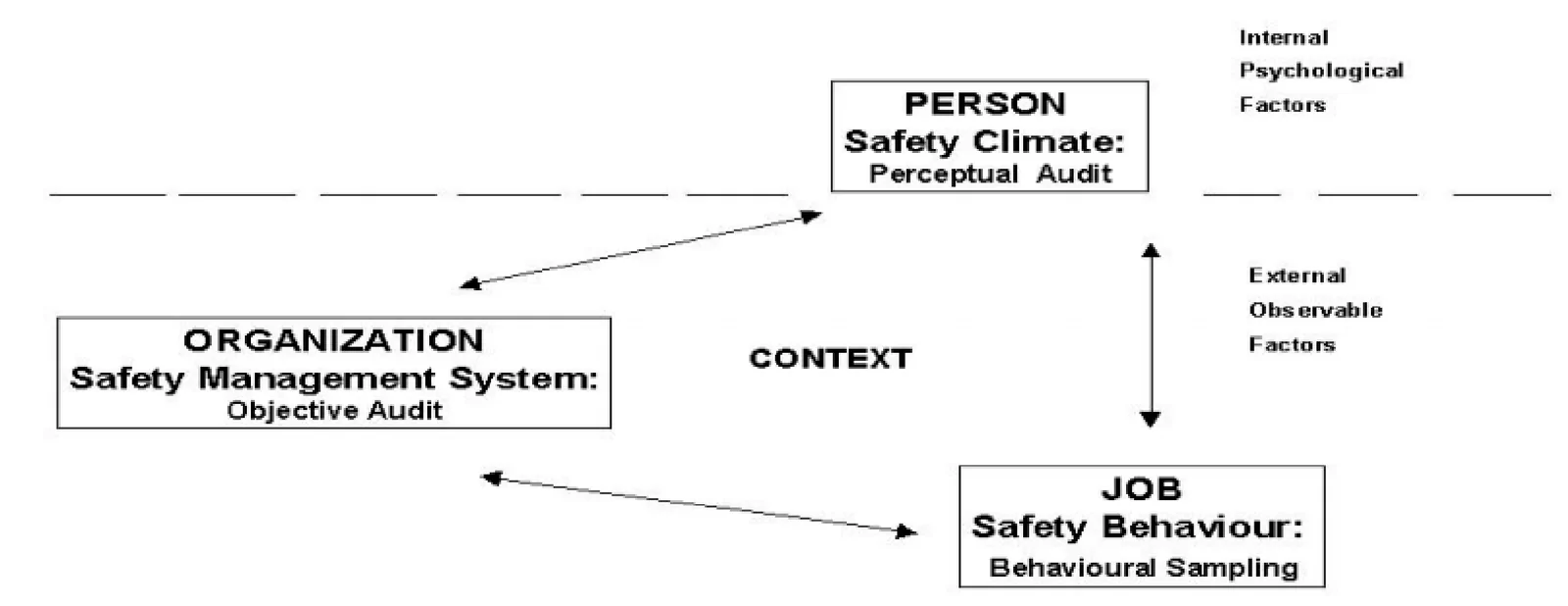
Reciprocal Safety Culture Model from Cooper (2000) [19].

Cooper interpreted safety culture through the lens of Bandura’s Model of Reciprocal Determinism [18,19]. This model describes interactions among psychological, behavioral, and situational components of safety culture [19]. The present study uses this model as an organizing framework rather than as evidence that the questionnaire comprehensively measures organizational safety culture. Attitudes are aligned with the psychological component, self-reported practices with the behavioral component, and reported work environment conditions with the situational component. Knowledge is included as a geriatric-specific cognitive capability within the individual domain; it is an extension of the framework for the present clinical context rather than a separate component explicitly proposed by Cooper (Figure 1).

Knowledge and practices are included because understanding geriatric safety principles and implementing safety-related actions are relevant to how safe care is supported and enacted. However, knowledge may function as an enabling factor, and practices may be both influenced by and contribute to safety culture. The cross-sectional design cannot establish these reciprocal or causal relationships. Consequently, the four subscales are interpreted as distinct, related dimensions relevant to geriatric patient safety culture, rather than equivalent measures of shared organizational culture.

Existing research on patient safety culture and geriatric care provides a foundation for this study [12,15,16,17,20]. However, general safety culture measures do not specifically integrate geriatric-focused nursing knowledge, attitudes, and safety practices with the work environment conditions relevant to intensive care. Addressing this assessment gap is important because organizational safety perceptions alone may not reveal gaps in nurses’ understanding or reported implementation of geriatric safety principles. The Geriatric Patient Safety Culture Questionnaire (GPSC-Q) was therefore developed to assess these four complementary domains among ICU nurses caring for critically ill older adults. By providing a domain-specific profile, the questionnaire may help distinguish educational needs from gaps in reported safety practices and work environment support, informing targeted improvement efforts while complementing established safety culture instruments.

### Study Aim

The study aimed to develop and psychometrically validate the Geriatric Patient Safety Culture Questionnaire (GPSC-Q) to assess ICU nurses’ knowledge, attitudes, self-reported practices, and perceptions of the work environment related to the safety of critically ill geriatric patients.

## 2. Methods

### 2.1. Study Design

This methodological study followed a three-phase approach to develop the Geriatric Patient Safety Culture Questionnaire (GPSC-Q). Phase 1 involved generating an initial item pool, Phase 2 focused on preliminary item evaluation, and Phase 3 assessed the questionnaire’s psychometric properties. The instrument encompasses four subscales: Knowledge, Attitude, Practice, and Work Environment.

### 2.2. Development of the GPSC-Q

Phase 1: Creating the item pool:

To develop the initial version of the scale, a comprehensive literature review was conducted using multiple databases, including Embase, MEDLINE, CINAHL, and PubMed. The search utilized a range of targeted keywords such as “geriatric critical care,” “pressure ulcers elderly ICU,” “older adult fall prevention,” “geriatric patient safety precautions and policies,” “sedation and pain management elderly ICU,” “age-related changes knowledge,” “older adults skin care,” “sleep management,” “urinary catheter care,” “geriatric patient restraint safety,” “nutrition and feeding of older adult,” “fall risk assessment,” “mental status assessment,” “patient privacy,” “geriatric ICU team collaboration,” “critical care decision-making elderly,” and “nursing training needs.” Based on the findings from this review, a preliminary pool of 128 items was created, organized into four subscales: nurses’ knowledge of safety culture for critically ill geriatric patients, nurses’ attitudes toward this safety culture, nurses’ practices to maintain it, and the work environment of the nursing staff in the ICU. The GPSC-Q was designed to assess individual and contextual dimensions relevant to geriatric patient safety culture. Knowledge and attitudes represent individual dimensions, practice captures self-reported safety behaviors, and work environment captures nurses’ reports of environmental and organizational conditions relevant to safe care. These subscales were developed with reference to dimensions addressed in prior studies [12,13,14,15,16,17,20,21]. The questionnaire items were formatted differently depending on the subscale: the first dimension used true, false, and do not know responses; the second and third used a 3-point Likert scale; and the fourth consisted of yes or no questions. For Knowledge items, correct responses were scored 1, whereas incorrect and “do not know” responses were scored 0; therefore, “do not know” responses were treated as an absence of demonstrated knowledge rather than missing data.

Phase 2: Preliminary Evaluation of the Items:

#### 2.2.1. Face Validity

Feedback on the initial scale was gathered from five critical care nurses, who assessed the items based on their clarity, relevance, ease of understanding, and overall appropriateness. Based on their input, 40 items were revised to correct wording issues, and an additional 10 items were modified to improve clarity and ensure their meaning was more easily understood.

#### 2.2.2. Expert Opinion

A panel of 15 academicians, including seven specialists in critical care nursing, six in geriatric nursing, and two experts in research methodology, evaluated the content validity of the scale using Lawshe’s method. Experts rated each item as essential, useful but not essential, or not necessary. The Content Validity Ratio (CVR) was calculated as CVR = (Ne − N/2)/(N/2), where Ne is the number of experts rating an item as essential and N is the total number of experts. For a panel of 15 experts, a minimum CVR of 0.60 was required for item retention [22]. Items with CVR values below 0.60 were removed.

#### 2.2.3. Pilot Study

A pilot study was conducted to determine whether the wording of the items was clear and easily understood. According to the existing literature, a sample size of 30 to 50 participants is considered adequate for pilot testing [23]. In this case, the 100-item draft questionnaire was administered to a group of 60 nurses who shared similar characteristics with the intended study population. Items were revised or removed based on pilot participants’ feedback regarding clarity, comprehensibility, ambiguity, and redundancy, resulting in an 80-item version. The 80-item version entering psychometric evaluation comprised 25 Knowledge, 14 Attitude, 24 Practice, and 17 Work Environment items. The 60 nurses who participated in the pilot study were excluded from the main psychometric evaluation sample.

#### 2.2.4. Participants

Nurses were recruited using a convenience sampling method from six intensive care units across the main University Hospital and the Emergency Hospital affiliated with Mansoura University Hospital in Dakahila Governorate, Egypt. These ICUs admit geriatric patients in the acute stages of various illnesses. To be included in the study, participants had to be at least 20 years old, of any gender, directly involved in caring for critically ill elderly patients, have worked in the mentioned ICUs for at least one month (to ensure adequate orientation to the unit), and express a willingness to participate. For scale development research, a sample size between 200 and 300 is considered optimal [24,25]. Comrey and Lee (2013) categorize sample sizes as follows: 50 (very poor), 100 (poor), 200 (fair), and 300 (good) [26]. In this study, the sample size met the criteria for developing a scale with solid psychometric quality. A total of 350 questionnaires were distributed, and 345 were returned. After excluding 15 surveys due to incomplete responses, the final analysis included data from 330 participants. Because the instrument comprised four conceptually distinct subscales, factor analyses were conducted separately for each subscale rather than on all 80 initial items simultaneously.

#### 2.2.5. Data Collection

This study was carried out across six intensive care units across the main University Hospital and the Emergency Hospital affiliated with Mansoura University Hospital in Dakahila Governorate, Egypt. These units cater to geriatric patients presenting with various acute health conditions. To develop the item pool for the questionnaire, expert input was collected from 15 academicians affiliated with different Egyptian universities using the Lawshe method. Data were gathered using a self-administered questionnaire designed to assess ICU nurses’ knowledge, attitudes, self-reported practices, and perceptions of the work environment related to the safety of critically ill geriatric patients. To maximize participation and accommodate nurses’ preferences, both printed and electronic formats of the questionnaire were made available. Before completing the survey, participants were thoroughly briefed on the study’s purpose and procedures, and written informed consent was obtained. Participation was entirely voluntary, with strong measures in place to ensure both anonymity and confidentiality.

Data collection occurred between August 2025 and January 2026, beginning after ethical approval was obtained on 13 August 2025. Throughout this period, the research team remained in contact with participants to offer clarification and address any questions about the questionnaire. The survey included the draft 80-item GPSC-Q, along with a section capturing nurses’ socio-demographic details such as age, gender, marital status, educational background, hospital and unit affiliation, years of general and ICU-specific nursing experience, work shifts, and any prior training related to patient safety. To maintain a strong response rate, reminder messages were sent out regularly throughout the data collection period.

#### 2.2.6. Psychometric Analysis

Phase 3. Evaluating the Psychometric Properties:

Data analysis was performed using SPSS Statistics, version 27, and IBM SPSS Amos, version 24.0 (IBM Corp., Armonk, NY, USA). The initial screening process involved checking for missing values, outliers, univariate and multivariate normality, and multicollinearity. Univariate outliers were identified through z-scores, with values exceeding +3 or falling below −3 flagged for further review. Univariate normality was assessed using skewness and kurtosis, with skewness values within ±3 considered acceptable [27] and kurtosis values within ±7 deemed suitable [28]. To evaluate multivariate normality, Mardia’s multivariate kurtosis coefficient was compared to a critical value calculated as *v* × (*v* + 2), where *v* represents the number of observed variables. Multivariate normality was assumed if the coefficient did not exceed this threshold [29]. Multicollinearity was assessed by examining tolerance and Variance Inflation Factor (VIF) values, ensuring that tolerance was above 0.10 and VIF values were below 10. Maximum Likelihood Estimation (MLE) was used for parameter estimation during confirmatory factor analysis (CFA). For categorical variables, results were reported as frequencies and percentages, while means and standard deviations were used to describe continuous variables. Pearson’s correlation coefficients were calculated to explore the relationships between latent domains, with statistical significance set at *p* < 0.05.

The psychometric evaluation of the GPSC-Q questionnaire involved a range of analytical methods. Content validity was assessed by examining floor and ceiling effects, specifically by calculating the percentage of respondents who selected the lowest or highest possible scores on the scale. If more than 15% of participants scored at either extreme, it indicated the presence of a floor or ceiling effect. To evaluate construct validity, both Exploratory Factor Analysis (EFA) and Confirmatory Factor Analysis (CFA) were conducted, along with assessments of convergent and discriminant validity.

The number of factors to retain in the model was determined using Kaiser’s criterion and visual inspection of the scree plot. According to Kaiser’s rule, only factors with eigenvalues greater than one were retained, while the scree plot was used to identify factors appearing above the “elbow” or inflection point, which were considered meaningful [30]. Given the objective of obtaining the best model fit, oblique rotation was used, as it allows for the possibility that factors may be correlated [27]. In this study, Promax, a commonly used oblique rotation method, was applied under the assumption that the extracted factors might not be entirely independent. Additionally, Principal Axis Factoring (PAF) was chosen as the extraction method. PAF operates within a probabilistic framework and is well-suited for psychometric research, as it emphasizes shared variance and aims to uncover latent constructs that explain the observed inter-item correlations [29].

To establish a stronger and more reliable factor structure, items with cross-loadings—defined as loadings above 0.30 on two or more factors with a difference in loadings of less than 0.1—were removed [31,32]. Additionally, any item with a factor loading below 0.40 was excluded from the scale [33]. Following this refinement, Confirmatory Factor Analysis (CFA) was carried out to validate the structure identified through Principal Axis Factoring (PAF). Parallel analysis was additionally conducted using tetrachoric or polychoric correlations, as appropriate to the item response format, to corroborate the number of factors retained for each subscale. Both the EFA and CFA were conducted using the same analytic sample of 330 participants. EFA was performed using IBM SPSS Statistics, version 27.0 (IBM Corp., Armonk, NY, USA), whereas CFA was conducted using IBM SPSS Amos, version 24.0 (IBM Corp., Armonk, NY, USA). The EFA was used to identify the preliminary factor structure of each subscale, and CFA was subsequently applied to examine the fit of the resulting measurement structure. An independent validation sample was not available in the present study; therefore, the CFA should be considered an internal confirmation of the factor structure rather than an independent cross-validation. Modification indices were reviewed to improve model fit by adding covariances between error terms when appropriate.

The overall model fit was assessed using several established indices, with the following thresholds used as criteria for acceptable fit: Comparative Fit Index (CFI) ≥ 0.95, Standardized Root Mean Square Residual (SRMR) < 0.08, Tucker–Lewis Index (TLI) > 0.95, Root Mean Square Error of Approximation (RMSEA) < 0.06, and chi-square to degrees of freedom ratio (χ^2^/df) < 5 [32].

As a sensitivity analysis, CFA was repeated using WLSMV with the binary and ordinal items specified as ordered categorical indicators. For the dichotomously scored Knowledge scale, KR-20 reliability, item difficulty (proportion correct), and corrected item-total discrimination indices were also calculated.

Convergent validity was assessed using the Average Variance Extracted (AVE), with values of ≥0.50 indicating that a construct explains at least 50% of the variance in its indicators. In addition, standardized factor loadings were examined, with loadings of 0.50 or higher considered supportive of convergent validity. Composite reliability (CR) was also examined to assess the internal consistency of the indicators representing each construct, with values of ≥0.70 generally considered acceptable. Convergent validity was primarily supported by an AVE value of ≥0.50, interpreted alongside the composite reliability estimates and the factor loadings of the indicators [28,34]. Discriminant validity was assessed by comparing the square root of the AVE for each construct with its correlations with other constructs. A construct was deemed to have adequate discriminant validity if the square root of its AVE exceeded its inter-construct correlation values [35].

Given the multidimensional structure and differing response formats, Knowledge, Attitude, Practice, and Work Environment scores were interpreted separately rather than combined into a single GPSC-Q total score. Higher Knowledge, Practice, and Work Environment scores indicate more favorable levels, whereas higher Attitude scores indicate poorer attitudes.

The internal consistency of the questionnaire was evaluated using item-total correlations and Cronbach’s alpha coefficients. A Cronbach’s alpha value of 0.70 or higher was considered the threshold for acceptable reliability.

#### 2.2.7. Ethical Considerations

The study was approved by the Research Ethics Committee of the Faculty of Nursing at Mansoura University (0798). Study participants received a detailed description of the study, and confidentiality was ensured for all participants. They were informed about the voluntary nature of their participation and assured that withdrawal would have no negative consequences. Before the study began, all participants provided written informed consent.

## 3. Results

The demographic data are presented in Table 1. A total of 330 nurses participated in the main study. The majority of ICU nurses in the study were young (mean age 27.82 years) with limited professional (<5 years, 60%) and ICU (<5 years, 64.6%) experience, which may impact their ability to implement safety culture effectively. While most hold a bachelor’s degree (58.2%), gaps in specialized training are evident, with 42.7% lacking general training, 81.8% untrained in risk management, and 60.9% without geriatric care training. Additionally, 80% worked rotating shifts, increasing the risk of fatigue-related safety issues.

Of the 80 items entering EFA, 37 were removed according to the prespecified factor-loading and cross-loading criteria. Specifically, 10 Knowledge, 7 Attitude, 12 Practice, and 8 Work Environment items were removed, resulting in a final 43-item questionnaire comprising 15 Knowledge, 7 Attitude, 12 Practice, and 9 Work Environment items “see more details in Supplementary Materials”.

The Exploratory Factor Analysis (EFA) of the Knowledge subscale identified four factors, explaining 76.33% of the variance, with a strong KMO value (0.884) indicating excellent sampling adequacy. Bartlett’s test of sphericity (χ^2^ = 4093.699, *p* < 0.001) confirmed significant item correlations, supporting the scale’s well-structured, multi-dimensional nature (Table 2).

The Attitude subscale (Table 3) exhibited a single-factor structure, accounting for 68.64% of the variance, with a high KMO value (0.885) ensuring strong sampling adequacy. Bartlett’s test (χ^2^ = 2467.244, *p* < 0.001) validated significant inter-item correlations. All seven items (ATT3, ATT4, ATT7, ATT5, ATT13, ATT6, and ATT8) loaded strongly (0.705–0.932), reinforcing their unidimensional nature and robust measurement properties.

The Practice subscale (Table 4) revealed four factors, explaining 64.44% of the variance, with a moderate KMO value (0.732). Bartlett’s test (χ^2^ = 1738.572, *p* < 0.001) supported factor extraction, highlighting the scale’s multidimensional nature, though refinements may enhance construct validity.

The Work Environment subscale (Table 5) extracted three factors, accounting for 47.24% of the variance, with a moderate KMO value (0.719). Bartlett’s test (χ^2^ = 682.120, *p* < 0.001) confirmed significant item correlations. The lower KMO suggests potential refinements and further validation to strengthen the factor structure.

Overall, the EFA results confirm distinct factor structures across the scales, with strong validity for knowledge and attitude measures, while practice and work environment scales may benefit from further refinement to optimize their psychometric properties. Parallel analysis supported four factors for Knowledge, four for Practice, and three for Work Environment, consistent with the original EFA solutions. For Attitude, parallel analysis suggested two factors rather than one. However, subsequent WLSMV CFA of this alternative two-factor model showed mixed fit (CFI = 0.998, TLI = 0.997, RMSEA = 0.197, SRMR = 0.022) and yielded a Heywood case, indicating that the alternative structure was not sufficiently stable to replace the original model.

Corrected item-total correlations ranged from 0.207 to 0.882 (Table 6). Although all retained items exceeded the prespecified minimum criterion of 0.20, W.env8 (r = 0.207), Pract2 (r = 0.256), and Pract16 (r = 0.282) showed relatively weak correlations. These items were retained because item retention considered both statistical and content relevance rather than item-total correlations alone; however, their performance warrants further evaluation in an independent sample.

In Table 7, internal consistency varied across the GPSC-Q components. The Knowledge subscale demonstrated high reliability across its domains (α = 0.855–0.943; total α = 0.902), while the Attitude subscale showed high internal consistency (α = 0.936). The Practice domains demonstrated acceptable to high reliability (α = 0.810–0.880), whereas the overall Practice coefficient was lower (α = 0.749). The Work Environment subscale showed comparatively weaker reliability, particularly for Domains 2 and 3 (α = 0.622 and 0.641, respectively). Although the overall 43-item coefficient was α = 0.833, this value should not be interpreted as evidence of adequate reliability for each component, which should be evaluated separately.

As displayed in Table 8, all Knowledge and Practice domains and the Attitude scale had AVE values above 0.50 and satisfactory CR values, supporting convergent validity. The Attitude scale showed CR = 0.938 and AVE = 0.687. For the Work Environment subscale, Domain 1 met the recommended criteria (CR = 0.754, AVE = 0.523), whereas Domains 2 and 3 showed lower CR (0.645 and 0.634) and AVE values (0.394 and 0.371), indicating weaker convergent validity. Discriminant validity was supported where the square root of AVE exceeded the corresponding inter-domain correlations, with values ranging from 0.818 to 0.893 for Knowledge, 0.775 to 0.847 for Practice, and 0.609 to 0.723 for Work Environment.

Inter-subscale correlations are presented in Table 9. Knowledge was positively correlated with Practice (r = 0.497, *p* < 0.001) and Work Environment (r = 0.293, *p* < 0.001), and negatively correlated with Attitude (r = −0.259, *p* < 0.001). Practice was positively correlated with Work Environment (r = 0.389, *p* < 0.001) and negatively correlated with Attitude (r = −0.205, *p* < 0.001). The correlation between Attitude and Work Environment was not statistically significant (r = −0.047, *p* = 0.393).

As displayed in Table 10, the model fit indices were assessed for the Knowledge, Attitude, Practice, Work Environment, and overall GPSC-Q scales before and after modifications.

**Figure 2:**
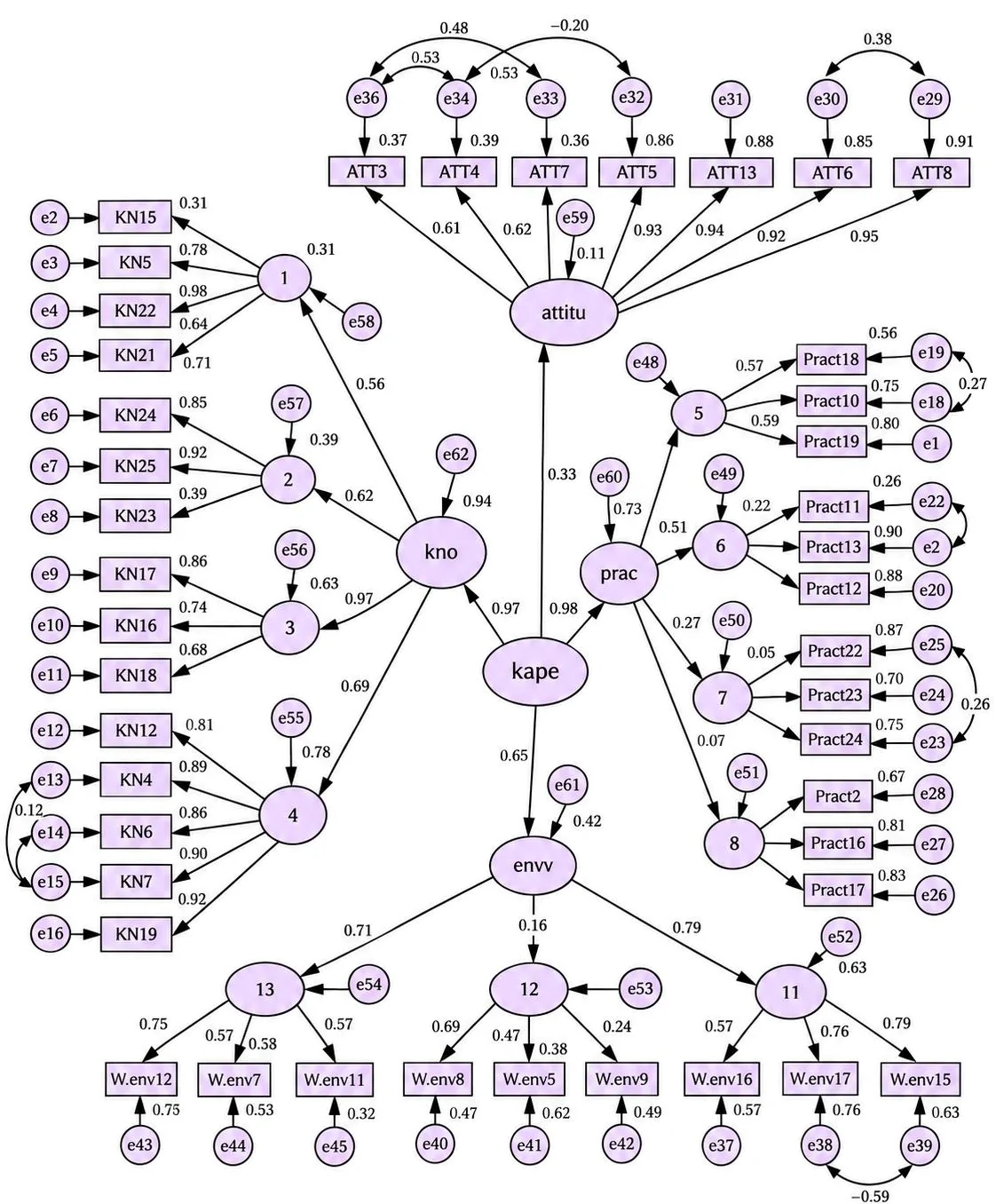
The second order for the GPSC-Q scale.

• Knowledge subscale: The initial model fit (CMIN/df = 2.131, RMSEA = 0.059, CFI = 0.977, TLI = 0.971) was acceptable, but modification indices improved fit (CMIN/df = 1.682, RMSEA = 0.046, CFI = 0.987, TLI = 0.982), suggesting a well-fitting model.
• Attitude subscale: The unmodified model exhibited poor fit (CMIN/df = 23.564, RMSEA = 0.262, CFI = 0.872). After modification, the model achieved substantial improvement (CMIN/df = 3.249, RMSEA = 0.083, CFI = 0.992, TLI = 0.981), ensuring an adequate representation of the data.
• Practice subscale: The initial model (CMIN/df = 1.504, RMSEA = 0.039, CFI = 0.980, TLI = 0.986) already displayed a good fit, with modifications further improving fit indices (CMIN/df = 1.171, RMSEA = 0.023, CFI = 0.996, TLI = 0.993).
• Work Environment subscale: The initial model showed poor fit (CMIN/df = 4.061, RMSEA = 0.096, CFI = 0.888, TLI = 0.832). Although modification improved the fit (CMIN/df = 3.634, RMSEA = 0.089, CFI = 0.907, TLI = 0.855), several indices remained below the prespecified criteria. Overall, the Work Environment subscale demonstrated weaker psychometric performance than the other subscales, particularly for Domains 2 and 3; therefore, its three-factor structure should be interpreted cautiously and requires further validation and potential refinement in an independent sample.
• Model refinement involved seven residual covariances in the Knowledge model, five in the Attitude model, four in the Practice model, and one in the Work Environment model, as displayed in Figure 2. These covariances were not prespecified but were introduced sequentially based on modification indices and substantive similarity between items.

As shown in Table 11, the categorical WLSMV sensitivity analysis supported the four-factor Knowledge and Practice structures, with high CFI and TLI values and low RMSEA and SRMR values. The modified one-factor Attitude model showed high CFI and TLI and a low SRMR, although the RMSEA remained elevated (0.135), indicating mixed fit. The Work Environment model also showed mixed fit (CFI = 0.946, TLI = 0.919, RMSEA = 0.088, SRMR = 0.103), although all standardized factor loadings were significant and ranged from 0.617 to 0.979.

Second-order CFA of the GPSC-Q:

A second-order CFA was conducted to examine the hierarchical structure of the GPSC-Q. The model showed mixed fit (χ^2^ = 1444.371, df = 828, CMIN/df = 1.744, CFI = 0.936, TLI = 0.930, RMSEA = 0.048, 90% CI = 0.043–0.052). The standardized higher-order loadings were 0.97 for Knowledge, 0.33 for Attitude, 0.98 for Practice, and 0.65 for Work Environment. Thus, Knowledge and Practice showed strong relationships with the higher-order construct, Work Environment showed a moderate relationship, and Attitude showed a comparatively weaker relationship. Given the CFI and TLI values below the prespecified 0.95 criterion, the higher-order structure should be interpreted cautiously (Table 10, Figure 2).

## 4. Discussion

As the care of critically ill geriatric patients becomes increasingly complex, there is a growing need for a targeted approach to ensuring safety in intensive care settings. In response to this need, the present study developed and evaluated a comprehensive questionnaire addressing nurses’ knowledge, attitudes, practices, and perceptions of the work environment related to the safety of critically ill geriatric patients. Rather than viewing awareness as a single construct, the findings suggest that geriatric patient safety awareness in intensive care is multidimensional and reflects both individual nursing capabilities and characteristics of the environment in which care is delivered. This interpretation is clinically important because deficits in one component may not necessarily be reflected in another, making domain-specific assessment potentially more informative than reliance on a single overall score [25,36].

The four-factor structure identified for the Knowledge subscale provides a clinically coherent representation of the knowledge required when caring for critically ill older adults. The domains Clinical Practices, Specialized Critical Care Procedures, Age-Related Physiological Changes, and Safety and Recovery Factors in Elderly Care suggest that geriatric ICU knowledge extends beyond general nursing knowledge [37]. It requires nurses to integrate age-related physiological vulnerability with technically complex critical care and safety-focused recovery practices. This multidimensionality is consistent with contemporary gerontological nursing frameworks in which competency incorporates clinical knowledge, age-specific assessment, safety considerations, and context-sensitive care rather than representing a single knowledge domain [37].

The knowledge structure may therefore have practical value for educational needs assessment. For example, a nurse may demonstrate adequate knowledge of routine clinical practices while requiring additional education concerning physiological changes associated with ageing or specialized critical care procedures. In this respect, domain profiles may help ICU educators identify specific learning needs and design more focused geriatric critical care education. However, such use should currently be regarded as an educational application requiring further validation rather than evidence that questionnaire-guided education itself improves patient safety.

The Attitude subscale demonstrated a comparatively coherent item set. However, the dimensionality of the Attitude subscale should be interpreted cautiously because the exploration and confirmatory analyses provided partially mixed evidence. Although a one-factor solution appeared more parsimonious, further validation in independent samples is warranted [25,36]. Although the exploratory analysis supported a single factor and internal consistency was high, parallel analysis suggested the possibility of two factors. The alternative two-factor solution was unstable, while the modified one-factor CFA produced mixed evidence of fit in the categorical sensitivity analysis. Thus, the findings favor retaining the more parsimonious one-factor interpretation at this stage, but they do not establish the Attitude structure definitively [38]. The need for several correlated residuals may also indicate overlap in wording or content among some attitude items. Independent cross-validation is therefore required before considering this structure fully established [25,36].

The four-factor Practice structure is also conceptually meaningful. Medication and Nutritional Safety, Psychological Support and Effective Communication, Technical Nursing Care, and Professional Safety and Quality Nursing Care reflect different ways in which nurses translate awareness into bedside care. Importantly, the emergence of psychological support and communication as a separate component indicates that safe geriatric critical care is not limited to technical procedures. Older critically ill patients may have communication difficulties, cognitive vulnerability, complex nutritional needs, and heightened dependence, making relational and technical aspects of nursing practice complementary rather than interchangeable.

The comparatively lower reliability of the overall Practice score is consistent with the multidimensional nature of this subscale, whereas the individual Practice domains demonstrated more satisfactory internal consistency. This pattern supports prioritizing domain-level interpretation over reliance on a single overall Practice score. Consequently, the domain scores may currently provide more clinically meaningful information than interpreting Practice as a single homogeneous construct. This is consistent with psychometric guidance emphasizing that high overall internal consistency should not be used as the sole justification for combining conceptually distinct dimensions [36,38].

The Work Environment subscale requires the greatest caution. Its three factors Specialized Policies and Procedures for Care, Work Environment, and Nursing Efficiency and Availability of Information for Care are clinically plausible because geriatric safety depends not only on nurses’ individual knowledge and practices but also on policies, information availability, staffing processes, and institutional support. Similar geriatric-care instruments have recognized the care environment as an important component of age-friendly nursing practice [39,40]. For example, the Geriatric Institutional Assessment Profile evaluates nurses’ knowledge, attitudes, and the institutional environment supporting geriatric care, emphasizing that age-friendly practice is partly an organizational property rather than solely an individual nurse characteristic [39].

Nevertheless, the Work Environment findings represent the principal psychometric weakness of the current questionnaire. Two domains demonstrated comparatively low internal consistency and inadequate convergent validity; several items showed relatively weak item-total relationships, and both conventional and categorical CFA produced only mixed model fit [38,41]. These findings suggest that the Work Environment domains are not yet as psychometrically stable as the Knowledge and Practice components. Accordingly, Domains 2 and 3 should not presently be treated as robust stand-alone measures for high-stakes comparisons, competency judgments, or organizational benchmarking. Further qualitative review, item refinement, and validation in an independent sample are warranted [25,38]. The relatively small number of items within each domain and the breadth of the organizational concepts being assessed may partly account for this weaker consistency [25].

The item-level findings reinforce this cautious interpretation. Although retained items met the prespecified minimum item-total criterion, the relatively weak performance of W.env8, Pract2, and Pract16 suggests that statistical retention does not necessarily establish optimal item functioning. Item decisions should balance psychometric performance against content coverage, because removing items solely to maximize reliability can narrow the construct being measured [36,42]. These items should therefore be specifically re-examined during subsequent validation, including cognitive interviewing with ICU nurses to determine whether wording, interpretation, or contextual relevance contributes to their weaker associations.

The convergent and discriminant validity findings generally support the distinction among Knowledge, Attitude, Practice, and Work Environment. However, the updated results do not support the previous statement that all domains achieved satisfactory convergent validity. In particular, the weaker Average Variance Extracted and Composite Reliability for two Work Environment domains confirm that these components require further development [37,43]. By contrast, the Knowledge and Practice domains and the Attitude scale showed stronger convergent evidence. This pattern illustrates why reliability, convergent validity, discriminant validity, and structural fit should be considered jointly rather than interpreting any single psychometric coefficient as sufficient evidence of validity [38].

The observed relationships among the major subscales also provide useful construct-level information. Knowledge was positively related to Practice and Work Environment, while Practice was positively related to Work Environment [42]. These associations are theoretically plausible because greater geriatric safety knowledge may coexist with stronger reported safety practices, while supportive organizational conditions may facilitate the enactment of those practices. Nevertheless, these correlations should not be interpreted causally [43,44]. The negative relationships involving Attitude reflect the reverse-scoring direction of that scale, where higher scores represent less favorable attitudes, rather than indicating that greater knowledge or better practice produces negative attitudes. The nonsignificant relationship between Attitude and Work Environment further suggests that nurses’ personal attitudes and their perceptions of organizational conditions may represent relatively distinct aspects of geriatric safety awareness [44,45].

The higher-order analysis provides an important qualification for interpretation of the total questionnaire score. Knowledge and Practice were strongly represented in the higher-order construct, whereas Work Environment showed a more moderate relationship and Attitude contributed comparatively less [25,37]. Together with the mixed global model fit, these findings suggest that the questionnaire should not yet be interpreted as measuring a fully homogeneous single construct. Accordingly, interpretation should prioritize subscale and domain scores rather than the overall score. Use of a total questionnaire score for benchmarking, classification, or evaluative purposes should await replication of the hierarchical structure and further evidence supporting the reliability and validity of the higher-order construct [46,47,48].

Compared with existing instruments, the proposed questionnaire appears to occupy a specific measurement niche rather than replacing established tools. Generic patient-safety instruments, such as the Hospital Survey on Patient Safety Culture and the Safety Attitudes Questionnaire, assess broad organizational safety culture but are not designed specifically around critically ill geriatric patients [49,50,51]. Gerontogeriatric competency instruments assess broader nursing competence for older adults, while the Geriatric Institutional Assessment Profile emphasizes age-friendly organizational care. Other ICU instruments focus on narrower issues such as delirium-related reflective competence rather than integrating knowledge, attitudes, practices, and work-environment factors within one geriatric critical-care framework [37,39,52]. Thus, the potential contribution of the present questionnaire lies in its simultaneous assessment of individual and contextual dimensions relevant to geriatric safety in intensive care, although comparative validity against established measures remains to be demonstrated.

From a clinical-practice perspective, these findings suggest several potential applications. ICU nurse educators could use domain-level results to identify areas requiring additional education, such as age-related physiological changes, medication and nutritional safety, communication with older critically ill patients, or knowledge of specialized policies [50]. Nurse managers could potentially use aggregated Work Environment responses to identify organizational issues requiring further assessment, such as access to information or geriatric-specific policies [37,49]. The questionnaire may also have future value for evaluating educational needs before and after geriatric-care programs. However, responsiveness to change has not yet been established, and the weaker Work Environment components require refinement before such applications become routine.

Importantly, the questionnaire should not currently be presented as demonstrating improved patient safety. The present study established preliminary psychometric evidence for measuring nurses’ awareness-related domains; it did not test whether questionnaire use changes nursing behavior, adverse events, or patient outcomes. Future studies should therefore examine test–retest reliability, measurement invariance across different ICU settings and nurse groups, responsiveness to educational interventions, criterion-related validity against relevant established instruments, and associations with objective indicators of geriatric nursing practice and patient safety [25,49,51]. Validation in independent and geographically diverse samples is particularly important because factor structures may vary across institutional and cultural contexts [25,52].

Overall, the findings provide preliminary psychometric support for the questionnaire as a multidimensional instrument, with comparatively strong internal support for the Knowledge and Practice structures and provisional support for the Attitude structure. However, the Work Environment domains and the higher-order total structure require further refinement and replication. At this stage, the instrument may be most appropriately used as a research and educational-needs assessment tool, with interpretation focused primarily on individual subscales and domains rather than whether every component or the total score has equivalent psychometric strength.

## 5. Limitations

This study contributes a geriatric-specific, multidimensional questionnaire and initial psychometric evidence for assessing dimensions relevant to patient safety among ICU nurses. Several limitations should nevertheless be considered when interpreting the findings. First, convenience sampling from six ICUs within two hospitals affiliated with the same university hospital system may limit generalizability. Although 330 nurses participated, the sample may not fully represent the diversity of professional backgrounds, organizational conditions, and nursing practices across other healthcare settings. Second, EFA and CFA were conducted using the same sample. Consequently, CFA provided an internal evaluation of the exploratory structure rather than independent confirmation. Residual covariances introduced through modification indices were also data-driven and may reflect sample-specific characteristics, requiring replication in an independent sample. Third, the primary factor analyses used methods that treated binary and ordinal responses as continuous. Supplementary CFA using WLSMV addressed the categorical response formats and provided additional evidence, but it does not resolve the limitations of the original exploratory analyses or replace independent validation.

Fourth, psychometric performance varied across components. The Work Environment subscale showed weaker model fit and lower internal consistency and convergent validity in two domains. The Attitude structure also warrants further examination because parallel analysis and confirmatory findings provided mixed evidence regarding dimensionality. These components require cautious interpretation and further refinement. Fifth, Attitude, Practice, and Work Environment responses were self-reported and may be affected by social desirability or recall bias. The assessed Knowledge items demonstrated knowledge rather than its application in clinical care. Individual-level responses also do not directly establish shared organizational safety culture, and the study did not include behavioral observations or safety-system audits. Its cross-sectional design precludes causal interpretation of relationships among components. Finally, test–retest reliability, known-groups validity, and comparison with established instruments were not assessed. Temporal stability and external construct validity therefore remain to be established. Future studies involving independent, diverse samples, repeated assessments, and complementary objective measures would extend the evidence supporting the GPSC-Q and clarify its practical applications.

## 6. Conclusions & Recommendations

This study developed the 43-item Geriatric Patient Safety Culture Questionnaire (GPSC-Q), integrating ICU nurses’ knowledge, attitudes, self-reported practices, and work environment conditions relevant to the safety of critically ill older adults. The questionnaire complements general safety culture instruments by incorporating geriatric-specific aspects of intensive care. Exploratory factor analysis identified four Knowledge factors, one Attitude factor, four Practice factors, and three Work Environment factors, providing a differentiated framework for examining these complementary dimensions.

The findings provide initial psychometric evidence, with strengths and areas for refinement across the questionnaire. Knowledge demonstrated high internal consistency and satisfactory structural fit. Practice showed satisfactory model fit and domain-level reliability, although its comparatively lower overall internal consistency and limited correlations between some domains support prioritizing domain-level interpretation. Attitude demonstrated high internal consistency, but mixed confirmatory findings and uncertainty regarding its dimensionality warrant further examination. Work Environment requires particular attention because two domains showed lower reliability and convergent validity, and model fit remained below several prespecified criteria.

These findings support interpreting the components and their constituent domains separately rather than combining them into a single GPSC-Q total score. Independent validation is a priority, particularly because exploratory and confirmatory analyses used the same sample and model refinement involved data-driven modifications. Further studies should examine weaker items and domains, assess test–retest reliability, evaluate known-groups validity using clearly characterized groups, and compare GPSC-Q scores with established measures of related constructs.

The GPSC-Q provides a basis for further research on dimensions relevant to geriatric patient safety in intensive care. Following additional validation, its potential to inform educational needs assessment and organizational improvement can be evaluated prospectively. Recommendations for specific training programs or claims of improved nursing performance and patient outcomes should be based on subsequent evidence concerning score interpretation and intervention effectiveness.

## Figures and Tables

**Table 1:** Demographic data (n = 330).

	No.	%
Age		
20–29	237	71.8
30–39	84	25.5
40–49	9	2.7
Mean ± SD	27.82 ± 4.5	
Sex		
Male	180	54.5
Female	150	45.5
Marital status		
Single	156	47.3
Married	156	47.3
Divorced	9	2.7
Separated	9	2.7
Education		
Nursing diploma	18	5.5
Nursing technician	117	35.5
Bachelor	192	58.2
Master	3	0.9
Profession experience		
<5	198	60.0
5–<10	51	15.5
≥10	81	24.6
Mean ± SD	6.4 ± 5.2	
ICU experience		
<5	213	64.6
5–<10	42	12.7
≥10	75	22.7
Mean ± SD	5.8 ± 5.1	
Shift		
Morning only	12	3.6
Long	45	13.6
Night only	9	2.7
Three shifts	264	80.0
Training on patients’ safety		
No	141	42.7
Yes	189	57.3
Risk management		
No	270	81.8
Yes	60	18.2
Training on geriatric patients’ care		
No	201	60.9
Yes	129	39.1

**Table 2:** Summary results of Exploratory Factor Analysis of knowledge subscale (N = 330).

Principal Axis Factoring, Rotation Method: Promax with Kaiser Normalization.						
Items	Factor 1	Factor 2	Factor 3	Factor 4	Eigen Value	% Variance
KN15	0.811				1.874	12.495
KN5	0.870					
KN22	0.885					
KN21	0.863					
KN24		0.947			6.222	41.480
KN25		0.922				
KN23		0.874				
KN17		0.826				
KN16			0.892		1.254	8.357
KN18			0.725			
KN12			0.827			
KN4				0.926	2.1	13.997
KN6				0.815		
KN7				0.926		
KN19				0.862		
Kaiser–Meyer–Olkin Measure of Sampling Adequacy = 0.884, Bartlett’s test of sphericity (χ^2^ = 4093.699; *p* < 0.001)						

Factor 1: Clinical Practices, Factor 2: Specialized Critical Care Procedures, Factor 3: Age-Related Physiological Changes, Factor 4: Safety and Recovery Factors in Elderly Care.

**Table 3:** Summary results of Exploratory Factor Analysis of Attitude subscale (N = 330).

Principal Axis Factoring, Rotation Method: Promax with Kaiser Normalization.			
Items	Factor 1	Eigen Value	% Variance
ATT3	0.705	4.805	68.643
ATT4	0.725		
ATT7	0.709		
ATT5	0.894		
ATT13	0.891		
ATT6	0.905		
ATT8	0.932		
Kaiser–Meyer–Olkin Measure of Sampling Adequacy = 0.885, Bartlett’s test of sphericity (χ^2^ = 2467.244; *p* < 0.001)			

Factor 1: Nurses’ attitudes toward geriatric patients’ safety culture.

**Table 4:** Summary results of Exploratory Factor Analysis of practice subscale (N = 330).

Principal Axis Factoring, Rotation Method: Promax with Kaiser Normalization.						
Items	Factor 1	Factor 2	Factor 3	Factor 4	Eigen Value	% Variance
Pract18	0.843				1.644	13.696
Pract10	0.688					
Pract19	0.795					
Pract11		0.852			2.975	24.788
Pract13		0.765				
Pract12		0.917				
Pract22			0.774		1.910	15.915
Pract23			0.803			
Pract24			0.815			
Pract2				0.658	1.205	10.040
Pract16				0.836		
Pract17				0.819		
Kaiser–Meyer–Olkin Measure of Sampling Adequacy = 0.732, Bartlett’s test of sphericity (χ^2^ = 1738.572; *p* < 0.001)						

Factor 1: Medication and Nutritional Safety, Factor 2: Psychological Support and Effective Communication, Factor 3: Technical Nursing Care, Factor 4: Professional Safety and Quality Nursing Care.

**Table 5:** Summary results of Exploratory Factor Analysis of work environment subscale (N = 330).

Principal Axis Factoring, Rotation Method: Promax with Kaiser Normalization.					
Items	Factor 1	Factor 2	Factor 3	Eigen Value	% Variance
W.env15	0.546			2.894	26.939
W.env17	0.583				
W.env16	0.965				
W.env9		0.442		1.172	7.706
W.env5		0.545			
W.env8		0.830			
W.env11			0.536	1.614	12.598
W.env7			0.549		
W.env12			0.724		
Kaiser–Meyer–Olkin Measure of Sampling Adequacy = 0.719, Bartlett’s test of sphericity (χ^2^ = 682.120; *p* < 0.001)					

Factor 1: Specialized Policies and Procedures for the Care, Factor 2: Work Environment, Factor 3: Nursing Efficiency and Availability of Information for the Care.

**Table 6:** Corrected Item-Total Correlation of knowledge, attitude, practice, and work environment subscales (n = 330).

Knowledge		Attitude		Practice		Work Environment	
Items	r	Items	r	Items	r	Items	r
KN15	0.536	ATT3	0.697	Pract18	0.429	W.env15	0.444
KN5	0.613	ATT4	0.719	Pract10	0.474	W.env17	0.518
KN22	0.609	ATT7	0.704	Pract19	0.393	W.env16	0.568
KN21	0.568	ATT5	0.848	Pract11	0.477	W.env9	0.300
KN24	0.648	ATT13	0.843	Pract13	0.381	W.env5	0.394
KN25	0.592	ATT6	0.857	Pract12	0.446	W.env8	0.207
KN23	0.658	ATT8	0.882	Pract22	0.384	W.env11	0.353
KN17	0.621			Pract23	0.316	W.env7	0.336
KN16	0.525			Pract24	0.424	W.env12	0.416
KN18	0.558			Pract2	0.256		
KN12	0.491			Pract16	0.282		
KN4	0.592			Pract17	0.348		
KN6	0.619						
KN7	0.597						
KN19	0.596						

**Table 7:** Internal Consistency of GPSC-Q questionnaire.

Domains/Scale	Items	Cronbach’s Alpha
Knowledge scale scores		
Domain 1	4	0.918
Domain 2	4	0.943
Domain 3	3	0.855
Domain 4	4	0.934
Knowledge total scores	15	0.902
Attitude scale scores		
Domain 1	7	0.936
Practice scale scores		
Domain 1	3	0.814
Domain 2	3	0.880
Domain 3	3	0.837
Domain 4	3	0.810
Practice total scores	12	0.749
Work environment scale scores		
Domain 1	3	0.761
Domain 2	3	0.622
Domain 3	3	0.641
Total work environment scores	9	0.721
Total GPSC-Q questionnaire scores	43	0.833

**Table 8:** CR, AVE, and correlation between the domains of Knowledge, Attitude, Practice, and Work Environment.

		AVE	CR	Dom 1	Dom 2	Dom 3	Dom 4
Knowledge subscale				r (*p*)	r (*p*)	R (*p*)	r (*p*)
	Domain 1	0.736	0.918				
	Domain 2	0.798	0.940	0.317 * (<0.001) *			
	Domain 3	0.669	0.857	0.363 * (<0.001) *	0.338 * (<0.001) *		
	Domain 4	0.780	0.934	0.324 * (<0.001) *	0.405 * (<0.001) *	0.415 * (<0.001) *	
	AVE square root			0.858	0.893	0.818	0.883
Attitude subscale		0.687	0.938				
Practice subscale				r (*p*)	r (*p*)	R (*p*)	r (*p*)
	Domain 1	0.605	0.820				
	Domain 2	0.717	0.883	0.212 (<0.001) *			
	Domain 3	0.636	0.840	0.135 (0.014) *	0.130 (0.019)		
	Domain 4	0.601	0.817	0.249 (<0.001) *	0.134 (0.015)	0.007 (0.901)	
	AVE square root			0.778	0.847	0.797	0.775
Work environment subscale				r (*p*)	r (*p*)	R (*p*)	r (*p*)
	Domain 1	0.523	0.754				
	Domain 2	0.394	0.645	0.212 (<0.001) *			
	Domain 3	0.371	0.634	0.413 (<0.001) *	0.142 (0.010)		
	AVE square root			0.723	0.628	0.609	

*: Statistically significant at *p* ≤ 0.05.

**Table 9:** Inter-subscale correlations among Knowledge, Attitude, Practice, and Work Environment (N = 330).

		Knowledge	Attitude	Practice	Work Environment
Knowledge	**r**				
	***p***				
Attitude	**r**	−0.259 *			
	***p***	<0.001 *			
Practice	**r**	0.497 *	−0.205 *		
	***p***	<0.001 *	<0.001 *		
Work Environment	**r**	0.293 *	−0.047	0.389 *	
	***p***	<0.001 *	0.393	<0.001 *	

r: Correlation: Pearson coefficient. *: Statistically significant at *p* ≤ 0.05.

**Table 10:** Model fit indices for Knowledge, Attitude, Practice, and Environment subscales, as well as the overall GPSC-Q scale.

Model	X2	df	CMIN/df	*p* Value	GFI	CFI	TLI	RMSEA	SRMR
Knowledge model without modification	178.967	84	2.131	<0.001	0.932	0.977	0.971	0.059	0.0399
knowledge model fit with modification indices	129.485	77	1.682	<0.001	0.950	0.987	0.982	0.046	0.0357
Attitude model without modification	329.093	14	23.564	0.001	0.774	0.872	0.808	0.262	0.105
Attitude model fit with modification indices	29.245	9	3.249	0.001	0976	0.992	0981	0.083	0.0139
practice model without modification	72.176	48	1.504	0.014	0.966	0.980	0.986	0.039	0.0377
Practice model fit with modification indices	51.544	44	1.171	0.203	0.975	0.996	0.993	0.023	0.0333
Work environment model without modification	97.463	24	4.061	<0.001	0.942	0.888	0.832	0.096	0.0689
Work environment model fit with modification indices	83.576	23	3.634	<0.001	0.952	0.907	0.855	0.089	0.0664
Second order of GPSC-Q scale	1444.371	828	1.744	<0.001	0.840	0.936	0.930	0.048	0.0704

**Table 11:** Sensitivity CFA using WLSMV for categorical indicators.

Scale	Factor Structure	Scaled χ^2^ (df)	CFI	TLI	RMSEA	SRMR	Standardized Loading Range
Knowledge	Four-factor	140.87 (85)	0.998	0.998	0.045	0.061	0.927–0.999
Attitude †	One-factor	63.05 (9)	0.999	0.998	0.135	0.013	0.692–0.977
Practice	Four-factor	64.81 (49)	0.997	0.996	0.033	0.050	0.805–0.959
Work Environment	Three-factor	84.47 (24)	0.946	0.919	0.088	0.103	0.617–0.979

† The Attitude sensitivity model included five correlated residuals corresponding to the modified primary CFA specification.

## Data Availability

The datasets used or analyzed in this study are available from the corresponding author upon request due to privacy concerns.

## Supplementary Materials

The following supporting information can be downloaded at: https://www.mdpi.com/article/10.3390/healthcare14203473/s1.
