# Supplementary material for: Development and Psychometric Validation of the Geriatric Patient Safety Culture Questionnaire for Intensive Care Nurses
Source: Healthcare (Basel). 2026 Oct 16;14(20):3473. doi: 10.3390/healthcare14203473 (PMC13704646; doi:10.3390/healthcare14203473)
Supplement: Supplementary file 1 [file healthcare-14-03473-s001.zip › healthcare-4593642-supplementary.pdf]

Geriatric Patient Safety Culture Questionnaire

GPSC-Q for intensive care nurses caring for critically ill older patients

Tool 1: Demographic and Occupational Characteristics of Critical Care Nurses

1. Name: ________________________________

2. Hospital: ______________________________

3. Unit: _________________________________

4. Age: __________

5. Sex: □ Male □ Female

6. Marital status: □ Single □ Married □ Divorced □ Widowed □ Separated

7. Educational qualification: □ Nursing diploma □ Technical nursing qualification □ Bachelor's degree in nursing □ Master's degree □ Other, please specify: __________________

8. How many years of experience do you have in this profession? __________

9. How many years have you worked in an intensive care unit? __________

10. Which shift do you work? □ Morning only □ Evening only □ Morning and evening (long shift) □ Night only □ Rotating among all three shifts

11. Have you received training in patient safety? □ No □ Yes

12. Do you participate in risk management committees? □ No □ Yes

13. Have you received training in the care of critically ill older patients? □ No □ Yes

Tool 2: Geriatric Patient Safety Culture Questionnaire

For intensive care nurses caring for critically ill older patients.

A. Knowledge

Select one response for each statement.

Item	Statement	True	False	Do not know
KN15	The symptoms of infection in critically ill older patients are the same as those in other patients.	◻	◻	◻
KN5	The diet of critically ill older patients is the same as that of other adult patients.	◻	◻	◻
KN22	Drug absorption, distribution, and elimination in older patients are the same as in other adult patients.	◻	◻	◻
KN21	It is preferable to use restraints for critically ill older patients.	◻	◻	◻
KN24	Ventilator settings (air volume and quantity, air pressure, and respiratory rate) for older patients differ from those for other patients in the intensive care unit.	◻	◻	◻
KN25	The ventilator humidification system and temperature settings for older patients differ from those for other patients in the intensive care unit.	◻	◻	◻
KN23	The doses of analgesics and sedatives for older patients receiving mechanical ventilation differ from those for other patients in the intensive care unit.	◻	◻	◻
KN17	It is preferable to use a urinary catheter only when necessary, in critically ill older patients who are conscious.	◻	◻	◻
KN16	Constipation is a common problem among critically ill older patients.	◻	◻	◻
KN18	Sensory function does not change with advancing age.	◻	◻	◻
KN12	The likelihood of developing hypertension decreases with advancing age.	◻	◻	◻
KN4	Wall grab bars, bed rails, and dry floors help critically ill older patients maintain their balance and prevent falls.	◻	◻	◻
KN6	Lung function is not affected by advancing age.	◻	◻	◻
KN7	Communicating with critically ill older patients in a calm and unhurried manner reduces their fear and anxiety.	◻	◻	◻
KN19	Older adults take longer to recover from physical and psychological stress.	◻	◻	◻

B. Attitude

Select one response for each statement.

Item	Statement	Agree (3)	Neutral (2)	Disagree (1)
ATT3	I prefer caring for younger patients to caring for critically ill older patients.	◻	◻	◻
ATT4	Critically ill older patients are very demanding.	◻	◻	◻
ATT7	Critically ill older patients can often provoke their caregivers, which may affect their safety.	◻	◻	◻
ATT5	Time should not be wasted following safety precautions for critically ill older patients with incurable diseases.	◻	◻	◻
ATT13	My personal problems can affect my performance in caring for older patients and maintaining their safety.	◻	◻	◻
ATT6	Critically ill older patients do not deserve the care and safety precautions provided by the healthcare team.	◻	◻	◻
ATT8	I feel stressed when caring for critically ill older patients and following safety procedures for them.	◻	◻	◻

C. Practice

Select one response for each statement.

Item	Statement	Always (2)	Sometimes (1)	Never (0)
Pract18	I know the precautions required when administering high-risk medications to older patients, such as heparin and insulin, and how to manage their adverse effects.	□	□	□
Pract10	I take into account the essential nutrients needed by critically ill older patients when feeding them orally or through a gastric tube.	□	□	□
Pract19	Hands are washed before and after contact with the older patient and their surroundings.	□	□	□
Pract11	I explain all nursing interventions to critically ill older patients before performing them.	□	□	□
Pract13	I reassure older patients in the intensive care unit.	□	□	□
Pract12	I listen attentively to conscious older patients in intensive care and meet their needs.	□	□	□
Pract22	When a suction device is used to remove secretions from older patients, its settings differ from those used for other patients in the intensive care unit.	□	□	□
Pract23	Different-sized connectors are used for older patients receiving mechanical ventilation compared with other patients in the intensive care unit.	□	□	□
Pract24	I reposition critically ill older patients before two hours have elapsed.	□	□	□
Pract2	I assess the health status of critically ill older patients during every shift.	□	□	□
Pract16	I maintain and respect the privacy of critically ill older patients in the intensive care unit.	□	□	□
Pract17	I always follow the five rights of medication administration when administering medications to critically ill older patients.	□	□	□

D. Work Environment

Select one response for each statement.

Item	Statement	Yes (1)	No (0)
W.env15	This unit has written policies and procedures for interventions, safety precautions, and the management of critically ill older patients.	☐	☐
W.env17	This unit holds awareness seminars on managing older patients receiving mechanical ventilation in the intensive care unit.	☐	☐
W.env16	This unit has written protocols for weaning older patients from mechanical ventilation that differ from those for other patients.	☐	☐
W.env9	Working in this hospital is like being part of a large family.	☐	☐
W.env5	The hospital where you work encourages personal development.	☐	☐
W.env8	I would feel safe being treated here as a patient.	☐	☐
W.env11	The number of nurses in this unit is sufficient to manage the number of patients.	☐	☐
W.env7	My colleagues encourage me to report any concerns I have about the safety of older patients.	☐	☐
W.env12	All information needed for diagnostic and treatment decisions concerning critically ill older patients is routinely available to me.	☐	☐

Subscale Scoring

- Knowledge: Score each correct answer 1 and each incorrect or "do not know" answer 0. Sum the 15 items; possible range 0- 15. Higher scores indicate greater knowledge.
- Attitude: Agree = 3, neutral = 2, and disagree = 1. Sum the 7 items; possible range 7-21. Higher scores indicate poorer attitudes.
- Practice: Always = 2, sometimes = 1, and never = 0. Sum the 12 items; possible range 0-24. Higher scores indicate more favorable self-reported practices.
- Work Environment: Yes = 1 and No = 0. Sum the 9 items; possible range 0-9. Higher scores indicate more favorable reports of work environment conditions.
- Interpret the four subscale scores separately. Do not combine them into a single questionnaire total score.
